# Strategies for consistent and automated quantification of HDL proteome using data-independent acquisition

**DOI:** 10.1016/j.jlr.2023.100397

**Published:** 2023-06-05

**Authors:** Douglas Ricardo Souza Junior, Amanda Ribeiro Martins Silva, Graziella Eliza Ronsein

**Affiliations:** Department of Biochemistry, Institute of Chemistry, University of São Paulo, São Paulo, Brazil

**Keywords:** apolipoproteins, data-independent acquisition, high-density lipoprotein, HDL, lipoproteins, proteomics, quantitative proteomics

## Abstract

The introduction of mass spectrometry-based proteomics has revolutionized the high-density lipoprotein (HDL) field, with the description, characterization, and implication of HDL-associated proteins in an array of pathologies. However, acquiring robust, reproducible data is still a challenge in the quantitative assessment of HDL proteome. Data-independent acquisition (DIA) is a mass spectrometry methodology that allows the acquisition of reproducible data, but data analysis remains a challenge in the field. To date, there is no consensus on how to process DIA-derived data for HDL proteomics. Here, we developed a pipeline aiming to standardize HDL proteome quantification. We optimized instrument parameters and compared the performance of four freely available, user-friendly software tools (DIA-NN, EncyclopeDIA, MaxDIA, and Skyline) in processing DIA data. Importantly, pooled samples were used as quality controls throughout our experimental setup. A careful evaluation of precision, linearity, and detection limits, first using *E. coli* background for HDL proteomics and second using HDL proteome and synthetic peptides, was undertaken. Finally, as a proof of concept, we employed our optimized and automated pipeline to quantify the proteome of HDL and apolipoprotein B–containing lipoproteins. Our results show that determination of precision is key to confidently and consistently quantifying HDL proteins. Taking this precaution, any of the available software tested here would be appropriate for quantification of HDL proteome, although their performance varied considerably.

## Graphical abstract

**Figure:**
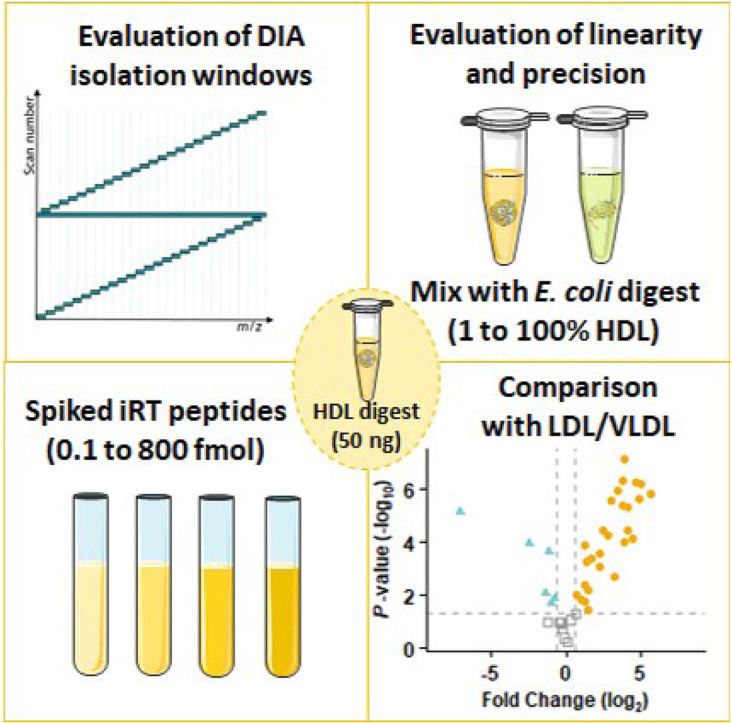

The role of high-density lipoprotein (HDL) as a protective risk factor for cardiovascular diseases is historically established (1). However, the disappointing clinical data obtained from raising HDL cholesterol levels in patients with coronary injury without any clinical benefit has highlighted the fact that the cholesterol content of HDL does not capture its complexity (2, 3). Since the publication of the first HDL proteomic study (4), HDL protein remodeling has been linked to cardiovascular outcomes. Interestingly, HDL proteome alterations have also been related to kidney diseases (5), diabetes (6, 7, 8), and recently even with the severity of COVID-19 (9). Therefore, precise quantification of HDL proteins may provide key information for metabolism-related diseases, and even for pathologies where HDL may play a previously unsuspected role.

Hundreds of proteins have been associated with HDL, but their adequate quantification remains a challenge (10, 11). A key issue is the fact that the majority of studies employed to quantify HDL proteins used an untargeted methodology, called data-dependent acquisition (DDA), or simply shotgun proteomics (12). However, the stochastic nature of this untargeted method makes it less reproducible, negatively impacting the accurate detection of low-abundant proteins (13). On the other hand, quantitative proteomics provides robust and sensitive protein quantification, alleviating the drawbacks of discovery proteomics (13, 14, 15). The gold standard method for targeted proteomics is the selected (or multiple) reaction monitoring (SRM or MRM) (16). This methodology provides robust and precise quantification although with a time-consuming method development as the trade-off (17).

An alternative targeted quantitative methodology for clinical proteomics is called parallel reaction monitoring (PRM). The advantage of PRM over SRM is the detection of all fragments in a single cycle, without the need for choosing the fragments before the acquisition. However, the experiment still requires previous knowledge of the precursor ions (18). We have shown PRM provides similar quantification performance when compared to SRM for HDL proteome (19).

Data-independent acquisition (DIA) is a methodology that aims to bridge discovery and targeted proteomics. In this approach, thousands of proteins may be detected without prior knowledge (like in DDA proteomics), and targeted data extraction can be performed after acquisition while still providing quantitative abilities similar to SRM or PRM analyses (20). The data acquisition involves the cyclical recording of consecutive MS1 scans followed by MS2 scans for all precursor ions in predetermined isolation windows (15). One of the advantages of DIA is the ability to reinterrogate the data without the need to repeat sample analysis. As a result, the accuracy of data may be improved through the removal of interferences, or additional quantitative information may be obtained on proteins that were not previously identified. Our previous results show DIA delivers quantitative analysis of HDL proteome without the extensive work needed to develop an SRM or PRM methodology (21). However, data processing remains a bottleneck in DIA proteomics, and up to date, there is no consensus on how to process DIA-derived data for HDL analyses, although some benchmarking for DIA processing tools has been done recently in the context of whole proteomes (22, 23, 24). Freely available software tools such as DIA-NN (25), EncyclopeDIA (26), MaxDIA (27) and Skyline (28) are continually improved and updated, but there is an urgent need to standardize data analysis.

In this work, we have compared the performance of four software tools available for DIA, namely, DIA-NN (25), EncyclopeDIA (26), MaxDIA (27) and Skyline (28). There are other excellent choices to analyze DIA-derived data, but we only included in our analysis freely available and user-friendly software. With that in mind, we first optimized key parameters in the mass spectrometer, followed by a systematic comparison of the software’s performance, regarding the ability to identify the proteins correctly and to quantify them consistently. A careful evaluation of precision and detection limits, first using *E. coli* background for HDL proteomics, and second using HDL proteome and synthetic peptides of known concentration, was then undertaken. Finally, the HDL proteome was compared to the proteome obtained by the isolation of apolipoprotein B (APOB)-containing lipoproteins (low-density and very low-density lipoproteins, LDL and VLDL, respectively). The results provided here may be used as a guideline to improve HDL proteome quantification, alleviating the lack of consistency that hampers the advances in the field.

## Materials and methods

### Materials

Potassium bromide, potassium phosphate, diethylenetriaminepentaacetic acid, sucrose, agar, tris, ammonium bicarbonate, sodium deoxycholate, trifluoroacetic acid (TFA), Luria-Bertani (LB) broth, formic acid and acetonitrile (MS grade) were purchased from Sigma-Aldrich (San Luis, USA). Dithiothreitol (DTT) and iodoacetamide (IAA) were purchased from Bio-Rad. Trypsin was purchased from Promega. Synthetic labeled peptides were purchased from Thermo Scientific. *Escherichia coli* BL21(DE3) was kindly provided by Professor Shaker Chuck Farah.

### Isolation of HDL and LDL/VLDL

Plasma samples from six apparently healthy donors were obtained from blood collected in EDTA-containing vacuum tubes. The Research Ethics Committee from Pharmaceutical Sciences, University of São Paulo approved the study (CAAE 60860016.5.0000.0067). The human studies reported in this work abide by the Declaration of Helsinki principles. HDL was isolated by two-step density ultracentrifugation (29). Briefly, plasma density was adjusted to 1.21 g/ml (from an assumed background density of 1.006 g/ml) with potassium bromide and spun at 120,000 rpm for 6 h (5°C) in a TLA 120.2 rotor using Optima MAX-XP ultracentrifuge (Beckman Coulter). Then, the top fraction containing all lipoproteins was collected and its density was adjusted to 1.063 g/ml with saline. Samples were spun again at 120,000 rpm for 2 h (5°C). The bottom fraction was collected and labeled as HDL. The top fraction was collected and labeled as LDL/VLDL. Samples were dialyzed against dialysis buffer (20 mM potassium phosphate pH 7.4, 0.1 mM diethylenetriaminepentaacetic acid, 5% m/v sucrose). Protein content was measured using the Bradford assay (Bio-Rad) according to the manufacturer’s instructions.

### Bacterial culture and protein extraction

*E. coli* BL21(DE3) was plated onto agar-containing LB broth and incubated for 24 h at 37°C (250 rpm agitation). A single colony was then added into 10 ml of LB broth and grown overnight at 37°C with 250 rpm agitation. The saturated bacterial culture was centrifuged at 5,000 rpm for 10 min (4°C). The cell pellets were washed three times with 50 mM tris buffer. Pellets were then resuspended in 350 μl extraction buffer (50 mM ammonium bicarbonate, 1% sodium deoxycholate, 20 mM DTT). Bacteria were inactivated by heating for 10 min at 95°C. Cells were pooled and lysed using sonication for 20 min at 30 s on/off cycles (40% amplitude) using SONICS Vibra-cell VCX 130. Lysed cells were centrifuged (14,000 rpm, 30 min, 4°C) to remove cell debris. The protein concentration was measured using the Bradford assay.

### Protein digestion

For each sample, 10 μg of protein were digested (21). Proteins were diluted in a total of 100 μl 0.2% sodium deoxycholate in 100 mM ammonium bicarbonate. Then, samples were reduced with the addition of 5 μl 0.1 M DTT for 1 h (37°C), alkylated with 3 μl of 0.5 M IAA for 30 min at room temperature (RT), and excess IAA was quenched using 2.5 μl of 0.1 M DTT (15 min, RT). Freshly prepared trypsin (0.1 μg/μl) was added to HDL proteins at a ratio of 1:40 (w:w). Digestion proceeded for 4 h (37°C) before a second trypsin aliquot (1:50, trypsin:protein) was added. Digestion was carried out overnight (37°C). All steps were performed with 400 rpm shaking. The reactions were stopped and surfactant was precipitated by adding 0.6% TFA and incubating for 30 min at 37°C. Samples were recovered after spinning tubes for 30 min at 14,000 rpm (4°C). Digested proteins were then desalted using the C18-StageTip protocol (30), vacuum dried, and stored at −80°C until MS analyses. Prior to MS injection, samples were solubilized in 0.1% formic acid.

### LC-MS/MS analyses

Peptides were separated using an Easy-nLC 1200 UHPLC system (Thermo Scientific). Digested samples (50 ng) were loaded onto a NanoViper trap column (C18, 3 μm, 75 μm × 20 mm, Thermo Scientific) with 8 μl of solvent A (0.1% formic acid in water) at 900 bar. The trapped peptides were eluted onto an Acclaim PepMap analytical column (C18, 2 μm, 75 μm × 150 mm, Thermo Scientific) at a flow rate of 300 nl/min. Separation of peptides was accomplished using a linear gradient of 5–28% of solvent B (0.1% formic acid in 80% acetonitrile) for 25 min, followed by another linear gradient of 28–40% of solvent B for 3 min. Solvent B was then increased to 95% in 4 min, followed by 12 min of this washing step, before re-equilibration of the system with solvent A prior to each injection. Mass spectrometry data were acquired using an Orbitrap Fusion Lumos Tribrid mass spectrometer with a Nanospray Flex NG ion source (Thermo Scientific). A lock mass of a polydimethylcyclosiloxane ion (*m/z* 445.12003) was maintained as an internal mass calibration for all MS analyses. All data were acquired in positive mode using Orbitrap as the mass analyzer.

### Data-dependent acquisition

For DDA analyses, an MS1 scan was followed by data-dependent MS2 scans in a 3-s maximum cycle time. Once selected for the MS2 scan, precursor ions were excluded for subsequent MS2 scans for 40 s. MS1 data were acquired in profile mode with a resolution of 120,000 (at *m/z* 200), intensity threshold target of 5 × 10^4^, maximum injection time of 50 ms, and monitored scan range of 350–1550 *m/z*. Data-dependent MS2 scans were acquired in centroid mode with a resolution of 30,000 (at *m/z* 200), maximum injection time of 54 ms, quadrupole isolation window of 1.2 *m/z*, normalized HCD collision energy of 30, and an automatic *m/z* scan range.

### Data-independent acquisition

For DIA analyses, MS2 scans were acquired within predefined staggered precursor isolation windows of 8 *m/z* or 24 *m/z*. For 8 *m/z* window acquisition scheme, resolution was set as 15,000 (at *m/z* 200), maximum injection time of 22 ms, and precursor scan range from 396 to 1000 *m/z* with 0.5 *m/z* margins. For 24 *m/z* windows acquisition scheme, resolution was set at 30,000 (at *m/z* 200), maximum injection time of 54 ms, and precursor scan range from 388 to 1000 *m/z* with 0.5 *m/z* margins. For both methods, data were acquired in centroid mode, with a scan range of fragments from 150 to 1650 *m/z*, normalized HCD collision energy of 30 and AGC target of 5 × 10^5^. Precursor isolation list is available in supplemental Table S1.

### Assessment of analytical parameters

For the experiment comparing DIA window acquisition schemes (8 *m/z* and 24 *m/z*), HDL from one individual was digested, and technical variability was assessed by injecting this digested sample 11 times intercalating both methods. For the experiment using *E. coli* proteome as a background for HDL peptides, a pooled HDL sample from six individuals was mixed with *E. coli* digest at known ratios (from 1% to 100% protein mass from HDL) and injected in triplicates. For the experiment using HDL as a background for labeled peptides with known concentration, standard curves containing a mix of fifteen iRT peptides (Pierce retention time calibration mixture, Thermo Scientific, supplemental Table S2) ranging from 0.125 to 800 fmol/μl were generated in an HDL background. Standard curves with labeled apolipoprotein peptides (supplemental Table S2) using the same concentration range were also built. Each concentration point of labeled peptides in the HDL background was injected in triplicate (1 μl). Finally, for the experiment comparing HDL with LDL/VLDL, independent lipoprotein fractions from six individuals were analyzed, and they were also used to construct a technical variability pool (injected eight times).

### Data processing and statistical analyses

DDA mass spectra were searched against the reviewed version of UniProt human database (20,383 entries), using the Andromeda search engine within the MaxQuant software package (v.2.0.3.0) (31), with variable methionine oxidation and fixed cysteine carbamidomethylation. Trypsin was selected as protease, with two missed cleavages allowed. For confident protein identification, we used as criteria the presence of a minimum of two peptides (and at least one unique peptide) per protein. The MaxQuant msms.txt result file was used as a library source for DIA experiments, and it was converted to other library formats using the EncyclopeDIA software (v.1.12.31, (26)). Protein symbols (all capital letters, not italicized) are based on UniProtKB/Swiss-Prot entry gene names, although UniProt protein IDs are also available in the supplemental Tables.

DIA data were analyzed using four freely available, user-friendly (i.e., presenting a graphical user interface) software tools: DIA-NN (v.1.8.1, (25)), EncyclopeDIA (v.1.12.31, (26)), MaxDIA within MaxQuant platform (v.2.0.3.0, (27)) and Skyline (v.21.2.0.45, (28)). For DIA-NN and EncyclopeDIA, the Thermo Scientific raw files were converted to mzML files using MSconvert (v.3.0.11781). For all software, trypsin was designated as a protease with two missed cleavages allowed, and up to six transitions were selected to quantify each peptide. For the experiment with different acquisition window schemes, proteins were considered present in the sample if quantified in at least 50% of technical replicates. In Skyline software, all integrated peaks were manually inspected to ensure correct peak detection and integration, except when performed in automated mode, in which a mProphet model was used without filtering q values.

In each software, the linearity of all quantified proteins was assessed from the determination coefficient (*R*^2^) of a quadratic curve. Precision was assessed determining the coefficient of variation (CV) among replicates. A paired *t* test was used for comparison between HDL and LDL/VLDL proteins, with Benjamini-Hochberg correction of *P*-values. Statistical analyses and construction of graphs were performed using R (v.4.2.1), except for the graphical abstract created using Servier Medical Art (https://smart.servier.com/).

We defined labeled peptides quantification range as the interval between the lower limit of quantification (LLOQ) and the upper limit of quantification (ULOQ), using a log_2_-transformed linear regression curve. The LLOQ and ULOQ were respectively defined as the lowest and highest concentration where the CV of triplicate injections was below 20%, with an average accuracy within 80–120% (19).

## Results

### Rationale and study design

Our experiments were designed to evaluate the performance of different software tools used for DIA processing and to establish a guideline for the analysis of HDL proteomics data. We have chosen four freely available software that are both user friendly (present a graphical user interface) and require little to no special skills to analyze proteomics data. Our goal is to make DIA an accessible tool for clinicians and other non-proteomics expert researchers in the HDL field that have no background in bioinformatics or proteomics. A general workflow for our analyses is represented in the graphical abstract. Briefly, we started the study by optimizing DIA isolation windows in the mass spectrometer. Then, we investigated DIA quantification capabilities using both library-based and discovery (library-free) approaches. With the best parameters at hand, we evaluated the analytical performance of four DIA software tools (DIA-NN, EncyclopeDIA, MaxDIA and Skyline) using two strategies. First, we diluted HDL in *E. coli* background proteome. Second, we diluted synthetic labeled peptides in HDL proteome. Finally, we compared HDL and LDL/VLDL fractions as proof of concept of our DIA pipeline.

### Optimization of MS parameters in the data analysis is crucial for DIA proteomics

At first, our experiments aimed to find the best MS parameters in our instrumentation for DIA experiments. Thus, we analyzed 11 technical replicates of an ultracentrifuge-isolated, digested HDL sample from one individual using two isolation window schemes for DIA: staggered acquisition windows of 8 *m/z* or 24 *m/z* (supplemental Table S1), as previously suggested (32). Staggered isolation windows are advantageous due to their improvement in precursor selectivity without loss of key acquisition parameters (33). We also used two strategies for data analysis. In the first one, we analyzed data employing a spectral library built from our DDA experiments (library mode, 70 proteins). A spectral library contains information about retention time and fragment ions at the MS2 level, and it is used to query peptides in the DIA samples (15, 34). In the second approach, we analyzed the same data using the discovery mode. Each software employs a specific strategy to build its in silico library, using the whole human proteome as the resource. For discovery mode, DIA-NN builds an in silico spectral library from a FASTA file using a deep neural network (25). EncyclopeDIA allows the use of Walnut, an optimized version of PECAN (PEptide-Centric ANalysis) algorithm (35), enabling data analysis directly from predicted fragment spectra (26). MaxDIA discovery mode employs *in silico*-generated spectral libraries from DeepMass:Prism (27). Finally, because Skyline requires the user to set the targeted peptides for data analyses, we did not run it on discovery mode. At first view, the discovery mode seems attractive because there is no need to build a library from DDA experiments, reducing time for data collection and analysis, but it may not be as sensitive as library-based approaches (36) and results in lower reproducibility (37).

**Fig. 1:**
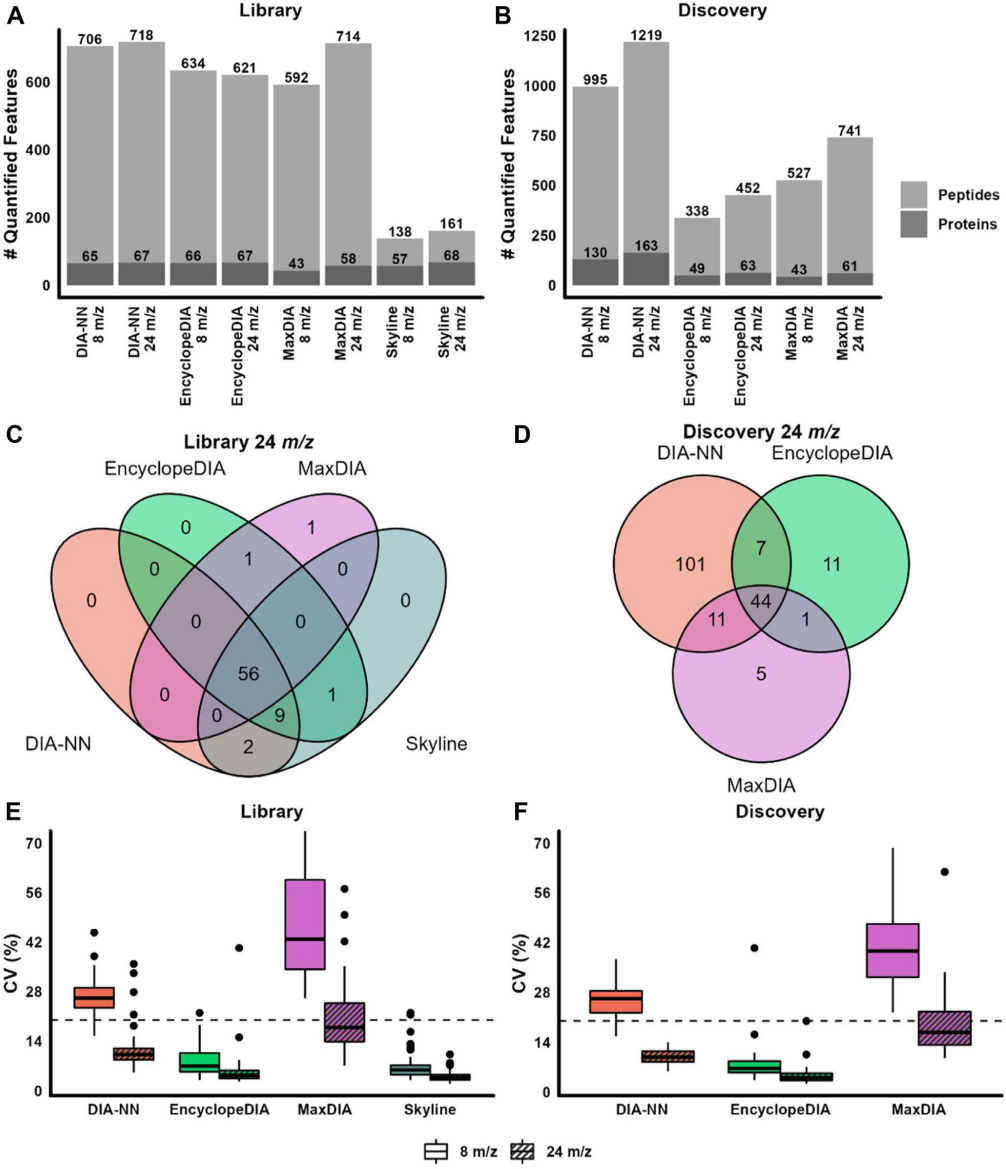
HDL proteome quantification using two staggered DIA window schemes (8 *m/z* and 24 *m/z*). HDL (50 ng) was analyzed using technical replicates (n = 11 per condition). A, B: Quantified features. The number of proteins and peptides quantified by each software tool in 8 *m/z* and 24 *m/z* DIA schemes is shown for library (A) and discovery (B) modes. To obtain those numbers, a library containing 70 proteins and 785 peptides was employed in (A), while all protein entries for the human proteome (n = 20,383) were considered for (B). C, D: Venn diagrams of proteins quantified by each software tool in library (C) and discovery (D) modes using the acquisition window of 24 *m/z*. E, F: Distribution of coefficients of variation (CVs) across 42 commonly quantified proteins. Plots were built for the library (E) and discovery (F) modes comparing the four software. For the sake of clarity, 11 data points with CV >70% were omitted from the library plot in (E)— one data point for EncyclopeDIA using 8 *m/z* window, one for MaxDIA using 24 *m/z* window, and nine data points for MaxDIA using 8 *m/z* window. The dashed line represents 20% CV.

Using the four software, we analyzed data obtained with 8 *m/z* and 24 *m/z* window acquisition schemes in library (Fig. 1A) and discovery (Fig. 1B) modes. Overall, the 8 *m/z* window scheme resulted in a similar or lower number of quantified proteins and peptides. In addition, for the three software that allowed both library and discovery modes (DIA-NN, EncyclopeDIA and MaxDIA), the library mode yielded a lower number of quantified proteins and peptides (Fig. 1A, B, respectively for library and discovery modes). Skyline had the lowest number of peptides and proteins due to the manual approach we employed to analyze the dataset, followed by MaxDIA, EncyclopeDIA, and DIA-NN. The latter displayed the highest number of quantified peptides and proteins, regardless of the mode the data was analyzed.

For the 24 *m/z* isolation window scheme using the library mode, the great majority of proteins were commonly quantified by all software tools (n = 56, Fig. 1C), with MaxDIA detecting the lowest number of proteins (n = 58, Fig. 1A). For the same isolation window scheme, but using the discovery mode, the software performed quite different from each other (Fig. 1D). DIA-NN found the highest number of proteins (163), but 68% of those proteins have never been described in HDL proteome (11), and therefore were considered false hits. EncyclopeDIA found 63 proteins using the discovery mode, 55 of them (87%) had previously been identified in the HDL proteome. Finally, MaxDIA discovery mode found 61 protein groups, with 59 hits amongst the previously detected in HDL proteome. Only 44 proteins were commonly quantified by the three data processing tools. The number of common proteins quantified in the library and discovery modes using the 8 *m/z* isolation window scheme was slightly smaller but comparable to that obtained with the 24 *m/z* scheme (supplemental Fig. S1A, B).

A key question that follows is the precision obtained by each software when quantifying HDL proteins. With that in mind, as a measure of precision, we selected only proteins quantified by all software in both isolation windows and analyzed the distribution of CVs for the 11 replicate injections using library (Fig. 1E, n = 42 proteins) and discovery (Fig. 1F, n = 35 proteins) modes. The distribution of CVs of all quantified proteins by each specific software is available in supplemental Fig. S1C–E. The 24 *m/z* isolation window scheme outperformed the 8 *m/z* one regardless of the software or mode analyzed, showing that the higher precision was related to the analytical performance of the mass spectrometer, not to the software utilized. For commonly quantified proteins, the MaxDIA software displayed the highest median CVs, independent of choosing library mode (median CV = 43% for 8 *m/z* and 17% for 24 *m/z*, Fig. 1E) or discovery mode (median CV = 40% for 8 *m/z* and 17% for 24 *m/z*, Fig. 1F). DIA-NN had relatively high CVs for 8 *m/z* windows in both library and discovery modes (median CV = 26% for both), but median CVs were considerably lower using the 24 *m/z* isolation window scheme (CV = 10% for both library and discovery modes). Of note, EncyclopeDIA displayed similar performance for common proteins for the analyses in library or discovery modes (median CV = 7% for 8 *m/z* and 4% for 24 *m/z*, Fig. 1E, F). As reported above, in Skyline software, peptides were analyzed using only library mode. Not surprisingly since data were manually curated, the later software displayed the lowest median CVs for 8 *m/z* (6%) and 24 *m/z* (<4%). When considering all quantified proteins, higher median CVs were displayed, independent of the software used for the quantification or the isolation window scheme tested (supplemental Fig. S1C–E).

Due to the high number of false hits obtained by some software tools in discovery mode, we continued our analyses using the library-based approach, even though the precision for common proteins was similar between both methods (Fig. 1C, D). A goal of this work was to provide a reliable and automated pipeline to analyze data from DIA experiments regarding HDL proteome. With that in mind, we also quantified the number of proteins with quantification CVs below 20% for each distinct software (Table 1). The 24 *m/z* isolation window scheme had the best performance, achieving the highest number of proteins with <20% CV. Manual curation using Skyline provided 98.5% of proteins with CV <20%, but was followed very closely by automated quantifications performed by DIA-NN (92.5%) and EncyclopeDIA (91%). We also compared precision for manual data curation and automated advanced peak picking algorithm using Skyline software. Our analysis shows manually curating data for Skyline provides better precision (lower CVs, supplemental Fig. S1E). The complete list of quantified proteins in 8 *m/z* and 24 *m/z* isolation schemes as well as the ones with CVs below 20% are displayed in supplemental Table S3. Because 24 *m/z* staggered acquisition window scheme had overall lower CV values, we continued with this scheme for the remaining of the analyses.

**Table 1:** Number of proteins with CVs below 20% obtained after quantification by each software tool

Software	Isolation Window	Quantified proteins (n)	Proteins with CV <20% (%)
DIA-NN	8 *m/z*	65	6.2
	24 *m/z*	67	92.5
EncyclopeDIA	8 *m/z*	66	75.8
	24 *m/z*	67	91.0
MaxDIA	8 *m/z*	43	0
	24 *m/z*	58	43.1
Skyline	8 *m/z*	57	94.7
	24 *m/z*	68	98.5

Taken together, our data showed a staggered 24 *m/z* scheme performed with overall lower CV values in our experimental conditions, with Skyline software (through manual curation) having the lowest CVs, closely followed by EncyclopeDIA and DIA-NN in library mode.

### Determination of precision is key when quantifying HDL-associated proteins

Studies have shown HDL protein cargo is altered in different pathophysiological states, with proteins considered minor components assuming important roles, despite their low abundance (38, 39, 40). Thus, in order to evaluate the abilities of the four software in quantifying HDL-associated proteins in a wide dynamic range, and in a complex matrix background, we mixed HDL and *E. coli* digests. Thus, 50 ng of digested proteins, ranging from 100% HDL (50 ng) to only 1% HDL (0.5 ng HDL), were analyzed. The list of proteins quantified by the four software tools in every HDL dilution is available in supplemental Table S4, and it is summarized in Table 2.

**Table 2:** HDL Proteins quantified at several dilutions in *E. coli* background

HDL	DIA-NN		EncyclopeDIA		MaxDIA		Skyline	
	Quantified proteins	With CV <20%^a^	Quantified proteins	With CV <20%^a^	Quantified proteins	With CV <20%^a^	Quantified proteins	With CV <20%^a^
1%	25	7	63	23	19	7	6	4
10%	41	28	61	29	28	10	13	12
25%	52	22	61	42	35	8	24	20
50%	73	46	62	51	36	19	50	48
100%	76	59	62	61	46	33	62	61

a CV was calculated only for proteins with 2 or 3 data points per HDL concentration.

In general, there is a progressive increase in the number of quantified proteins as the injected amount of HDL increases, as expected. Overall good performance was observed at 100% proteins from HDL, with all software quantifying at least 70% of proteins with CV below 20%. EncyclopeDIA and Skyline had similar performances at 100% HDL, both of them quantifying 61 out of 62 proteins with high precision (98.4%), followed by DIA-NN (59 out of 76, 77.6%) and MaxDIA (33 out of 46, 52.8%). Importantly, DIA-NN quantified the majority of proteins (76, with 59 of them below 20%), which is comparable to EncyclopeDIA and Skyline in absolute numbers of proteins quantified with high precision. MaxDIA had the lowest number of quantified proteins, with only 46 at 100% HDL.

At 50% HDL, we observed a slight reduction in the number of quantified proteins compared to pure HDL (reductions of 4%, 19% and 22% for DIA-NN, Skyline, and MaxDIA, respectively), except for EncyclopeDIA, which quantified a similar number of proteins (ranging between 61 and 63) for all tested HDL concentrations. The number of quantified proteins with low CV (<20%) decreased at 50% HDL, and was similar between DIA-NN (46 proteins), EncyclopeDIA (51 proteins), and Skyline (48 proteins). For lower HDL concentrations (<25% HDL), we observed a similar trend, but the number of quantified proteins was further reduced.

**Fig. 2:**
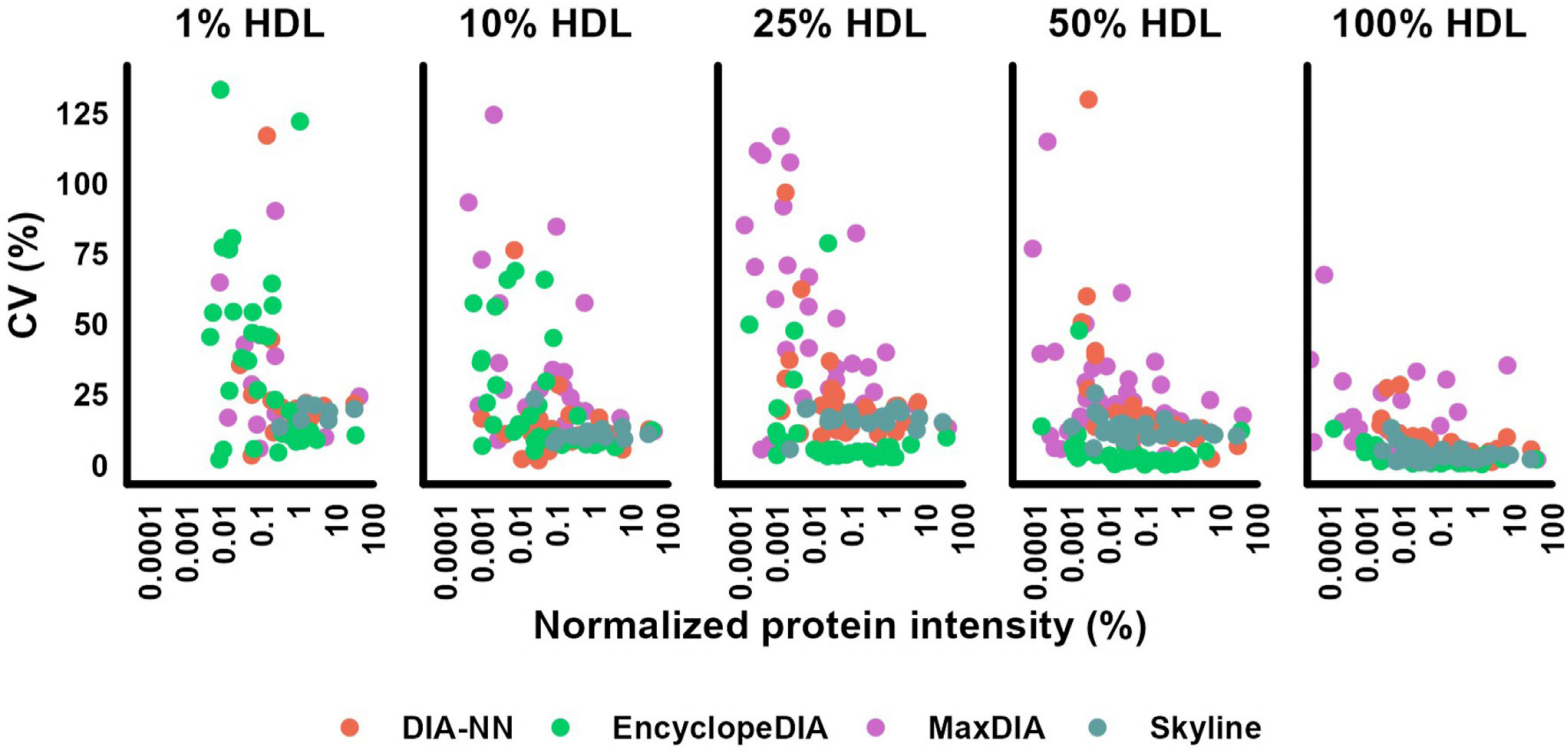
Precision of HDL quantification using *E. coli* as a background proteome. Pooled HDL digest from six individuals was diluted in *E. coli* digest, ranging from 1% to 100% HDL in 50 ng of total digested protein. The scatterplots show data from 37 HDL-associated proteins quantified by all software tools. In the y-axis, the CVs from triplicate injections are displayed. The mean intensity of each protein normalized for the total HDL intensity is displayed on the x-axis. The x-axis was log_10_-scaled for ease of data visualization.

Using scatterplots for data visualization, we represented 37 commonly quantified proteins across all four software, using their CVs, percent HDL dilution, and mean intensity (normalized as a percentage of the total intensity of each HDL dilution) (Fig. 2). In general, precision was lower (higher CVs) not only for proteins with low intensities (i.e., low-abundant proteins), but also for more diluted HDL samples. Low-intensity proteins were not quantified at lower HDL concentrations. Scatterplots for all proteins are available in supplemental Fig. S2.

**Fig. 3:**
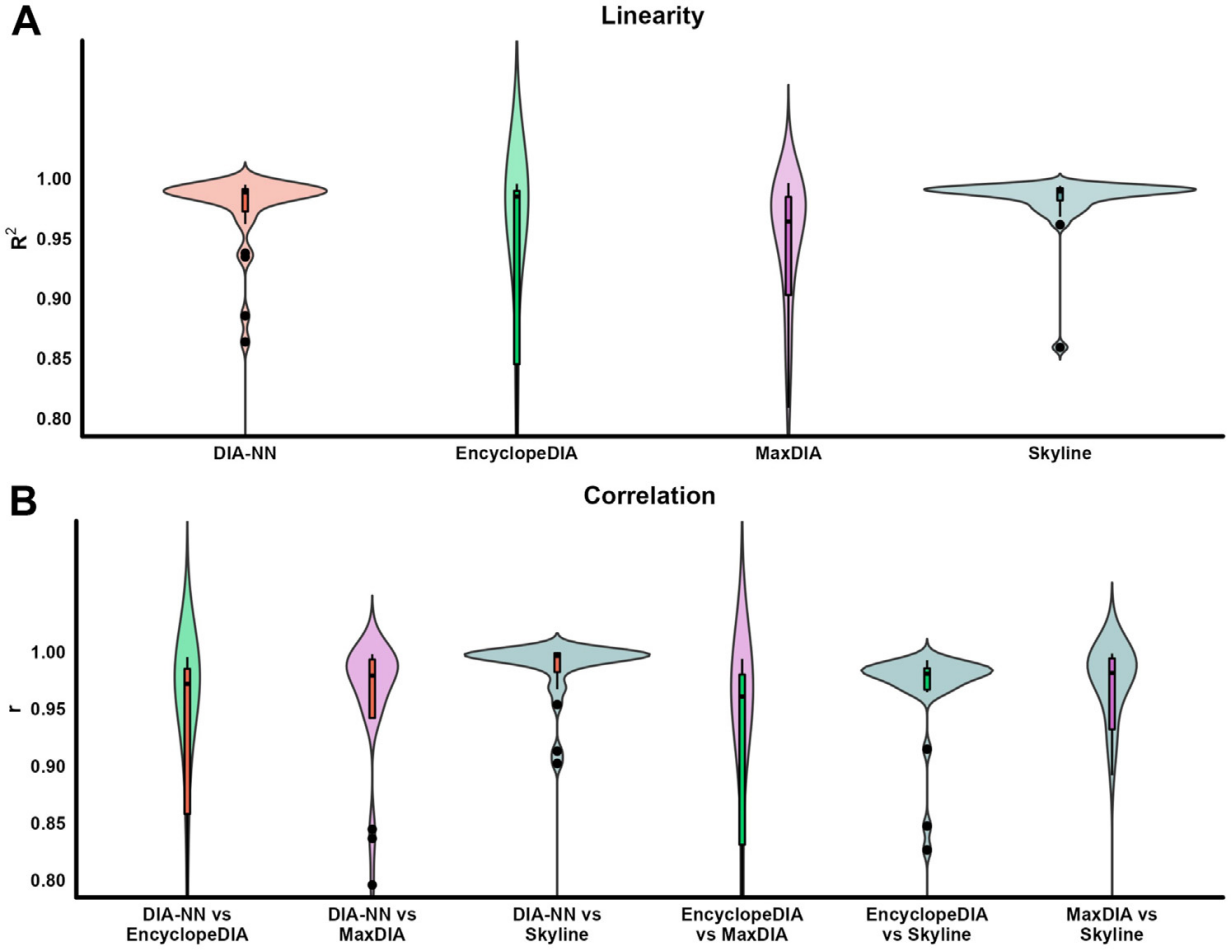
Assessment of linearity and correlation of HDL proteins diluted in *E. coli*. Pooled HDL from six individuals was diluted in *E. coli* digest, ranging from 1% to 100% HDL in a total of 50 ng. The plots show 33 proteins commonly quantified in two or more concentrations of HDL in at least one technical replicate across all four software platforms. A: We evaluated linearity using the determination coefficient (*R*^2^) of a quadratic curve. We omitted 14 data points with *R*^2^ below 0.8 from the plot (2, 8 and 4 data points for DIA-NN, EncyclopeDIA and MaxDIA, respectively). B: Distribution of Pearson correlation coefficients (r). We omitted 32 data points with *r* below 0.8 (7, 6, 3, 8, 3, and 5 data points for the comparisons DIA-NN vs. EncyclopeDIA, DIA-NN vs. MaxDIA, DIA-NN vs. Skyline, EncyclopeDIA vs. MaxDIA, EncyclopeDIA vs. Skyline and MaxDIA vs. Skyline, respectively).

Using *E. coli* proteome as a matrix background, we also assessed linearity and correlation using different software. Linearity was assessed by building a standard curve for each commonly quantified HDL protein (n = 33). Thus, the increasing concentrations of HDL proteins (1–100%) in *E. coli* background HDL were used to obtain the determination coefficients (*R*^2^) of quadratic function (Fig. 3A). The software tools displayed similar linearity, with most of the *R*^2^ above 0.8, although EncyclopeDIA and MaxDIA showed some skewness to lower *R*^2^. The correlation was evaluated using Pearson correlation of log_2_-transformed data (Fig. 3B). All six correlation pairs presented high correlation coefficients, with medians above 0.95, even though the comparison pairs DIA-NN versus Skyline, and EncyclopeDIA versus Skyline provided the narrower box plots, with the majority of r >0.95. Data for linearity (n = 79) and precision for all proteins (n = 65) are reported in supplemental Fig. S3. Individual plots for every HDL protein at an increasing concentration in the *E.coli* background are available in supplemental Fig. S4.

We selected APOA1 and APOA2, the two most abundant proteins in HDL to exemplify how differences in software performance could influence the obtained results. The four software quantified both proteins in triplicate injections across all replicates even in concentrations as low as 1% HDL in *E. coli* background. All software quantified APOA1 precisely in pure HDL (CV < 6% for all), and quantification remained robust (CV < 20%) down to 10% HDL. At 1% HDL, only EncyclopeDIA and Skyline could quantify APOA1 and APOA2 with CV <20%. APOA1 and APOA2 make up for about 70% and 20% of total HDL protein content, respectively (41). However, the HDL Proteome Watch reports 251 proteins likely pertaining to HDL (11), thus many less abundant proteins belong to HDL. These proteins are likely important for HDL function, but their precise quantification in low abundance may be a challenge. By using a complex matrix background (e.g., *E. coli* digest) and spiking in different amounts of digested HDL proteins, we can estimate the quantification capabilities of different software platforms. Thus, EncyclopeDIA and Skyline were able to quantify precisely approximately 0.2% of total digested protein (considering that APOA2 makes up to 20% of total HDL protein and that 1% of HDL proteins were added in *E. coli* digest).

With the advance of MS technologies, the ability to detect proteins in minute concentration raised considerably (42). However, the results obtained in this work highlight the importance of evaluating the CVs of replicate injections obtained for all HDL proteins, and not only the number of protein hits obtained. The determination of CVs for replicate injections should be performed before quantifying a set of clinical samples. This step is key to ensuring the precision of the quantifications. Overall, DIA-NN, EncyclopeDIA and Skyline displayed comparable performance in precisely quantifying HDL of proteins with CV <20%.

**Fig. 4:**
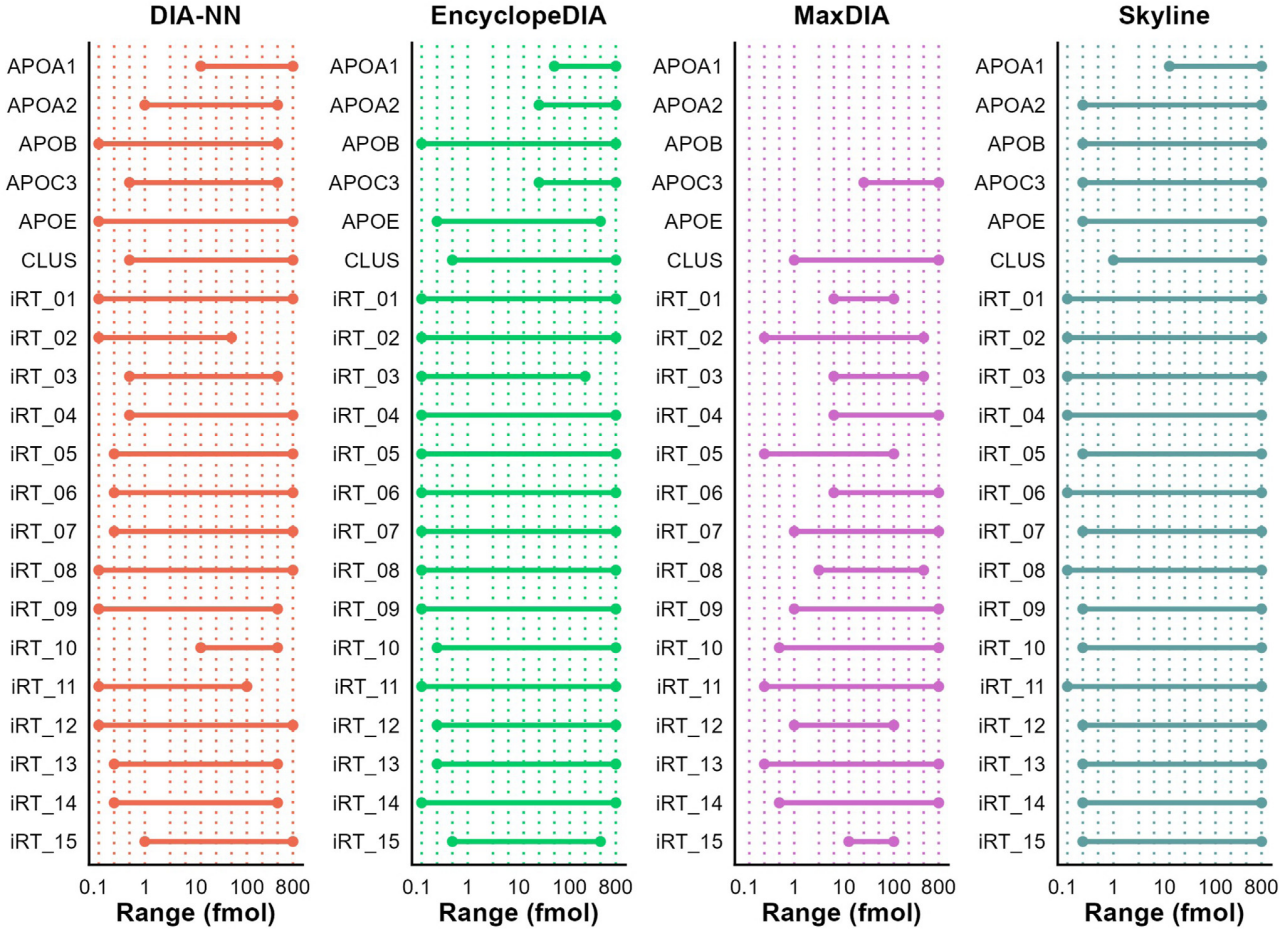
Quantification range of labeled peptides obtained by each software platform. Synthetic-labeled peptides (commercially available or specific for HDL proteins) of known concentrations (supplemental Table 2) were employed in the analyses. Synthetic peptides were diluted in HDL and quantified in triplicate. For each injection, 50 ng HDL containing 0.125–800 fmol peptides were analyzed. We defined labeled peptides’ quantification range as the interval between the lower limit of quantification (LLOQ) and the upper limit of quantification (ULOQ), using a log_2_-transformed linear regression curve. The LLOQ and ULOQ were, respectively, defined as the lowest and highest concentration where the CV of triplicate injections was below 20%, with an average accuracy within 80% to 120%. The x-axis was log_10_-scaled for ease of data visualization.

The experiments performed diluting HDL in *E. coli* background showed that even in a complex matrix, the software tools were able to distinguish and precisely quantify the majority of HDL proteins even with 50% of dilution. Since the quantifications provided are relative, and therefore do not determine the concentration of each protein detected, a key question that remained was regarding the sensitivity of DIA methodology. To answer this question, we used 50 ng HDL digest as a background to spike-in synthetic labeled peptides (supplemental Table S2) ranging from 0.125 to 800 fmols. For every peptide, we defined a quantification range as described in the Materials and methods section (Fig. 4). Although the dynamic range varied according to the software tool employed for quantification, a linear response for all labeled peptides was achieved without changes in HDL protein abundances, as measured by some peptides from common HDL proteins (supplemental Fig. S5). For all 21 quantified peptides, EncyclopeDIA and Skyline had the widest dynamic ranges, while MaxDIA had the narrowest ranges, mainly due to low precision of the quantifications (individual plots for each peptide are available in supplemental Fig. S6). Importantly, for the majority of peptides, DIA-NN, EncyclopeDIA, and Skyline tools were able to quantify with confidence (CV < 20%) as low as 250 amols of labeled peptides in complex HDL background (Fig. 4).

### DIA differentiates HDL and LDL/VLDL proteomes

**Fig. 5:**
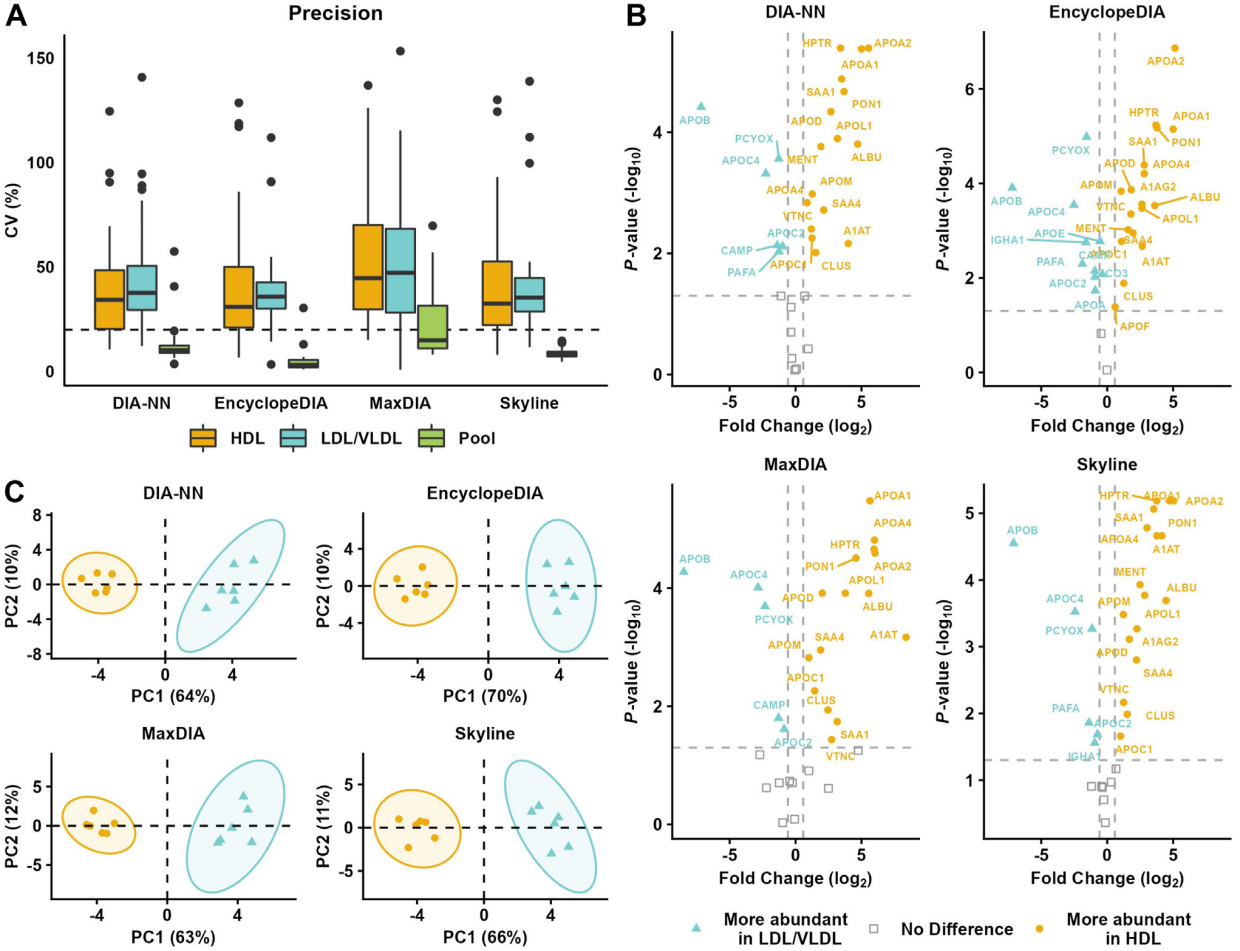
Comparative analysis of the proteome of lipoprotein classes (HDL and LDL/VLDL). HDL and LDL/VLDL were isolated from six apparently healthy individuals by two-step density ultracentrifugation and analyzed by DIA using 24 *m/z* staggered precursor isolation window scheme. For comparison, a pooled sample was constructed from equal parts of each fraction from every individual and injected eight times. We used the four software to compare the precision (A) and ability to differentiate both classes of lipoproteins (B) for 30 proteins commonly quantified by all four software. A: Distribution of CVs in boxplots. Pooled HDL sample was used to assess technical variability. B: Volcano plots of HDL and LDL/VLDL samples (statistical significance (−log_10_) in the y-axis and biological difference (log_2_ fold change) in the x-axis). C: PCA plots of HDL and LDL/VLDL fractions. A total of 27 commonly quantified proteins with less than 50% missing values in HDL or LDL/VLDL were used for PCA construction. Missing values were imputed with mean values from the other replicates (0.85% of values used for PCA were imputed).

Lipoproteins share a considerable amount of their protein cargo, but a specific protein may play different roles in the metabolism depending on where it is primarily located at a given time. For instance, HDL-containing APOC3 has been shown to be associated with a higher risk of coronary artery disease (43). Thus, quantification of the relative abundance of proteins in different lipoproteins may provide valuable information regarding metabolic or cardiovascular status. Therefore, the ability to compare lipoproteins’ proteome was assessed by isolating HDL (1.21 g/cm³ > density > 1.063 g/ml) and LDL/VLDL (density < 1.063 g/ml) fractions from the plasma of six individuals, and analyzing their proteome using DIA methodology, injecting the same amount of protein content for each fraction. A pooled sample consisting of equal amounts of each fraction was also analyzed to evaluate technical variability (n = 8 injections). The distribution of CVs for the 30 proteins quantified in both lipoprotein fractions using the four software tools (DIA-NN, EncyclopeDIA, MaxDIA, and Skyline) is shown in Fig. 5A. A list of all proteins quantified in every replicate for the lipoproteins’ fractions is available in supplemental Table S5. Both HDL and LDL/VLDL fractions exhibited similar median CVs, showing high biological variability (n = 6 subjects). As expected, those CVs were much higher than the ones obtained for the pooled sample, which measures only technical variability. The technical variability had median CV of 10%, 3%, 15%, and 8%, respectively, for DIA-NN, EncyclopeDIA, MaxDIA, and Skyline, contrasting with median CVs for biological variation considering both lipoprotein fractions of approximately 35% (similar for DIA-NN, EncyclopeDIA, and Skyline, except MaxDIA, which exhibited CV ∼45%). EncyclopeDIA presented the most reproducible results, even if we consider all quantified proteins (supplemental Fig. S7).

All four software tools yielded similar results when comparing lipoproteins’ proteome (Fig. 5B). Of note, MaxDIA exhibited overall larger fold changes than the other software, but the reasons for this large discrepancy are unknown.

Importantly, for all data analyses steps, we did not perform any data normalization. MaxQuant software (including MaxDIA mode) offers the popular MaxLFQ algorithm (44). DIA-NN can also perform MaxLFQ data normalization, as well as its own cross-run total ion intensity normalization (25). Data is automatically normalized by total ion count in the EncyclopeDIA software. One can disable this normalization step using the command line, but for simplicity, we used the default configuration. Finally, Skyline allows to skip normalization or to use equalized median or total ion count as ways of normalization. A previous study suggested DIA may not benefit from normalization in all datasets and performs best with non-normalized data (22). In our dataset, relatively low variability was achieved for non-normalized data obtained using all four software, but further investigation on different normalization techniques in the HDL field is encouraged.

Finally, all the software were able to differentiate the lipoproteins using PCA plots (Fig. 5C), with the first two principal components explaining more than 70% of the total variability. These results showed technical variability is much lower than biological variability for all evaluated software, and demonstrate the applicability of DIA methodology to evaluate relative differences in lipoproteins’ composition.

## Discussion

HDL is a dynamic and heterogeneous class of lipoprotein particles composed of several associated proteins presented in a wide dynamic range (10, 11). HDL proteins have been related to lipid metabolism but also inflammation and immune system (10, 11), making HDL proteomics an excellent choice to look for diseases-related mechanisms and potential biomarkers. However, the perspective of new findings through HDL proteomics is hampered by the lack of consistency in the analyses. Herein, we developed a precise pipeline for HDL proteomics using the DIA strategy. Instrument parameters were optimized and four user-friendly and freely available software tools were systematically evaluated. Although all four software were able to provide quantification results distinguishing HDL from LDL/VLDL fractions, they did not perform similarly regarding key analytical aspects.

First, using the optimized instrument parameters, our results showed that manual curation with Skyline was able to quantify the highest number of proteins with low CV (n = 67, CV < 20%) in a pooled HDL sample injected multiple times. Automated analyses using DIA-NN and EncyclopeDIA tools had similar performances (n = 62 and 61 proteins with CV < 20%, respectively), and MaxDIA had the lowest number of proteins quantified with high precision (n = 25). Indeed, the ICH (International Council for Harmonization of Technical Requirements for Pharmaceuticals for Human Use) M10 Bioanalytical Method Validation guideline recommends up to 20% CVs at the LLOQ for chromatographic methods. This recommended CV is even lower than the one recommended for ligand binding assays (up to 25% at LLOQ or ULOQ). Therefore, in this work, the majority of the proteins analyzed by at least three different software tools fall within the required precision. Using DIA proteomics, many peptidoforms were also described in HDL, and their characterization and quantification may be relevant in certain diseases (45). Although we did not search for peptidoforms, we recognize their relevance in HDL context, and further studies are required to evaluate the extent to which each software tool is capable of quantifying HDL modifications.

Since quantifications in lipoproteins’ proteome are not absolute, we also employed another strategy to determine the dynamic range (the lowest and highest quantification limits) using distinct software tools. We used synthetic labeled peptides (both, HDL-specific and commercially available) of known concentration diluted in digested HDL background. The results showed that Skyline, EncyclopeDIA, and DIA-NN were able to precisely distinguish and quantify as low as 250 amols of the synthetic peptides in the presence of 50 ng of digested HDL proteins as a matrix background. Both tools also provided linear results for quantifications up to 800 fmols of injected peptides, representing about four orders of magnitude, which is compatible with DIA quantification (15). MaxDIA showed the narrowest ranges for the peptides, due to low precision (CV > 20% for triplicate injection). Of note, MaxDIA is a relatively new implementation of MaxQuant software (27). Data processing was performed using MaxQuant v.2.0.3, but the development team has since made some improvements with the newer releases since v.2.1.4, although we did not test it.

When comparing HDL with LDL/VLDL, we used a pooled sample to measure technical variability from each software. This is important because the knowledge of the technical variance of a given protein allows us to judge if it can be quantified with precision in the specific dataset. Thus, we suggest that one should acquire data in a pool of samples whenever possible.

Isolation is a major source of variability in the HDL field, as previously shown (10, 11, 46) and, therefore, interferes with proteome quantification. Direct measurement of apolipoproteins’ concentrations from plasma would be ideal from a clinical standpoint to reduce variability due to HDL isolation, as previously performed by research groups (47, 48, 49). However, these two types of analyses may provide different information. It is key to point out that lipoproteins share proteins. For example, PLTP exists in an active form when isolated in the HDL size range from plasma, while PLTP associated with other lipoprotein classes are mainly inactive (50); also, high levels of HDL-containing APOC3 were associated with a decrease in insulin sensitivity (51). Furthermore, some proteins, such as clusterin, may possess a lipoprotein-binding fraction and a second lipid-free plasma portion (52).

Strengths of this work include a careful evaluation of several analytical parameters using different software, creating a pipeline to confidently quantify HDL proteome. Our work also has some limitations. First, as we aimed to have comparable software configurations, we did not try to optimize specific parameters for each software. However, specific parameters’ optimization could improve their analytical performance. Also, we did not compare commercial software tools, but we acknowledge their extensive use in the DIA literature (36). Finally, our conclusions cannot be generalized to the field outside of lipoprotein and HDL proteomics, since their complexity is different from that of cellular proteome or whole plasma, for example.

Based on our results, we hope researchers could make an informed decision about how to employ DIA for quantitative HDL proteomics with the best performance possible. A suggested strategy would be first to build a library with DDA data (pooling samples may be a time and cost-saving strategy). Whenever possible, library data should be collected close in time to minimize MS variability. Second, we recommend an optimization of MS parameters (isolation window scheme, for instance). We hope the data collected here may serve as a starting point for a quick optimization. Third, we advocate for a careful evaluation of precision for the proteins detected in the library. A good option would be to evaluate the CVs of triplicate injections of a pooled sample (a quality control). Proteins inconsistently quantified in the quality control sample (high CVs) should not be considered for clinical sample quantification, although it may be of interest to point out their presence in certain disease states. Importantly, we believe the variation of quantified proteins in quality control samples should always be reported. Taking these precautions, any of the available software platforms tested here would be appropriate for precise quantification of HDL proteome. The user may choose from manually curating data using Skyline software (aiming for best sensitivity and precision), or be aware of some compromise for high-throughput analysis, but still being able to trust the data due to known (low) variation in precision. Alternatively, a mixed strategy choosing a handful of proteins for Skyline manual validation may be appropriate. In the future, it will be important to employ labeled peptides or proteins for the quantification of HDL proteins.

## Data availability

The data supporting the findings of this study are available from the corresponding author upon reasonable request.

## Supplemental data

This article contains supplemental data.

## Conflict of interest

The authors declare that they have no known competing financial interests or personal relationships that could have appeared to influence the work reported in this paper.
