# Supplementary material for: Strategies for consistent and automated quantification of HDL proteome using data-independent acquisition
Source: J Lipid Res. 2023 Jun 5;64(7):100397. doi: 10.1016/j.jlr.2023.100397 (PMC10339053; doi:10.1016/j.jlr.2023.100397)
Supplement: Supplemental Figures S1–S7 [file mmc1.pdf]

# Strategies for consistent and automated quantification of HDL proteome using data-independent acquisition (DIA)

Douglas Ricardo Souza Junior[1], Amanda Ribeiro Martins Silva[1], Graziella Eliza Ronsein[1]*

## Supplemental Material

[1]Department of Biochemistry, Institute of Chemistry, University of São Paulo, São Paulo, Brazil

*Corresponding author

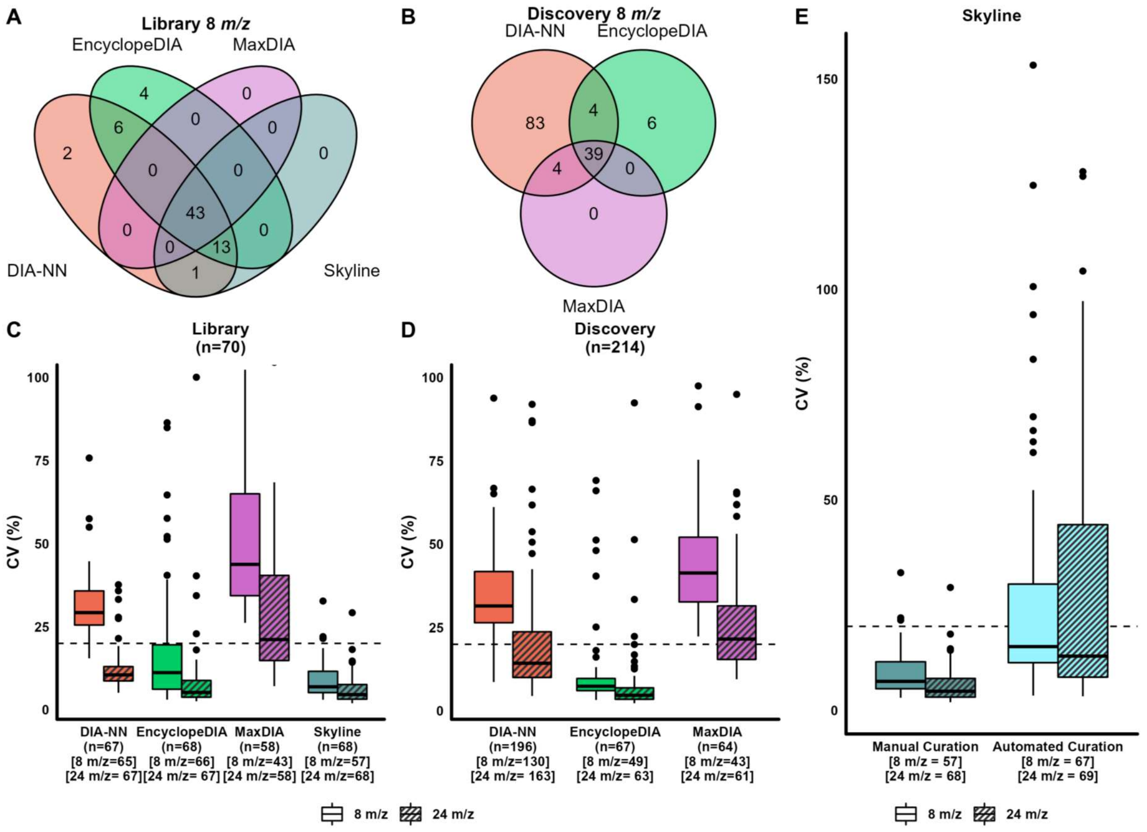


**Supplemental Figure S1. Precision of HDL quantification using two staggered DIA window schemes (8 *m/z* and 24 *m/z*).** HDL (50 ng) was injected in technical replicates (n=11 replicates each condition). The number of quantified proteins is identified in the plots. (A-B) Venn diagrams of proteins quantified in library (A) and discovery (B) modes of each software using acquisition windows of 8 *m/z*. (C-D) Distribution of CVs across all quantified proteins for library (C) and discovery (D) modes. Twelve data points with CVs higher than 100% were omitted from the plots. In the library mode plot (C), eight data points were omitted: CV of 135% for EncyclopeDIA 8 *m/z*, CVs of 100 and 178% for EncyclopeDIA 24 *m/z,* CVs of 100, 102 and 246% for MaxDIA 8 *m/z* and CVs of 105 and 296% for MaxDIA 24 *m/z*. In the discovery mode plot (D), four data points were omitted: CVs of 134 and 270% for DIA-NN 24 *m/z*, CV of 135% for EncyclopeDIA 24 *m/z* and CV of 247% for MaxDIA 24 *m/z*. (E) Distribution of CVs across all quantified proteins for Skyline software using manual curation or automated mProphet algorithm.

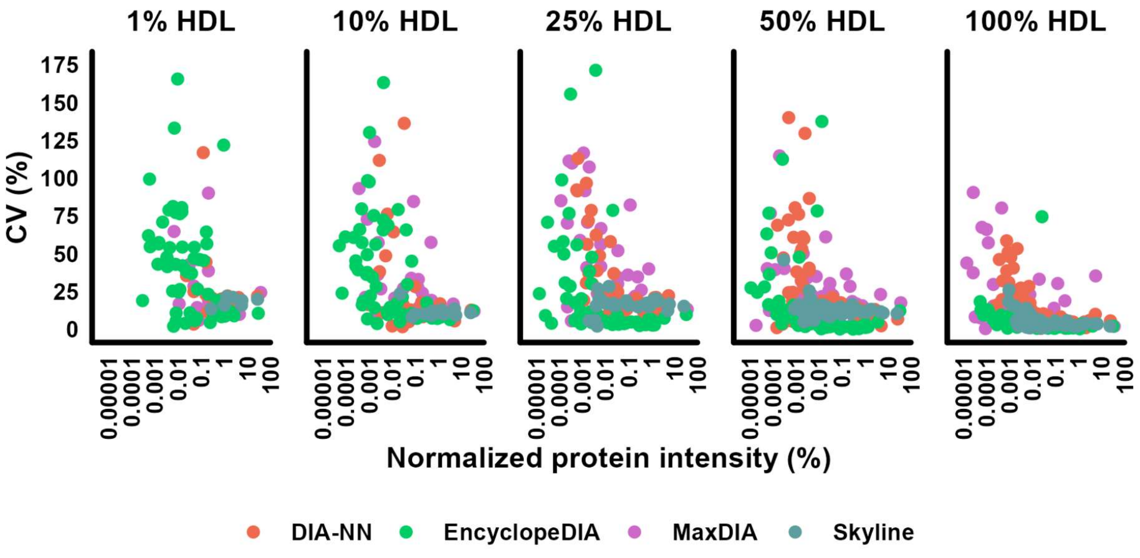


**Supplemental Figure S2. Precision of HDL quantification using *E. coli* as a background proteome.** Pooled HDL digest from six individuals was diluted in *E. coli* digest, ranging from 1% to 100% HDL in 50 ng of total protein. The scatterplots show data from 84 HDL-associated proteins quantified by the four software tools (76 for DIA-NN, 46 for MaxDIA and 62 for both EncyclopeDIA and Skyline). In the y-axis, the CVs from triplicate injections are displayed. The mean intensity of each protein (triplicate injections) normalized for the total HDL intensity is displayed on the x-axis. The x-axis was $log_{10}$-scaled for ease of data visualization.

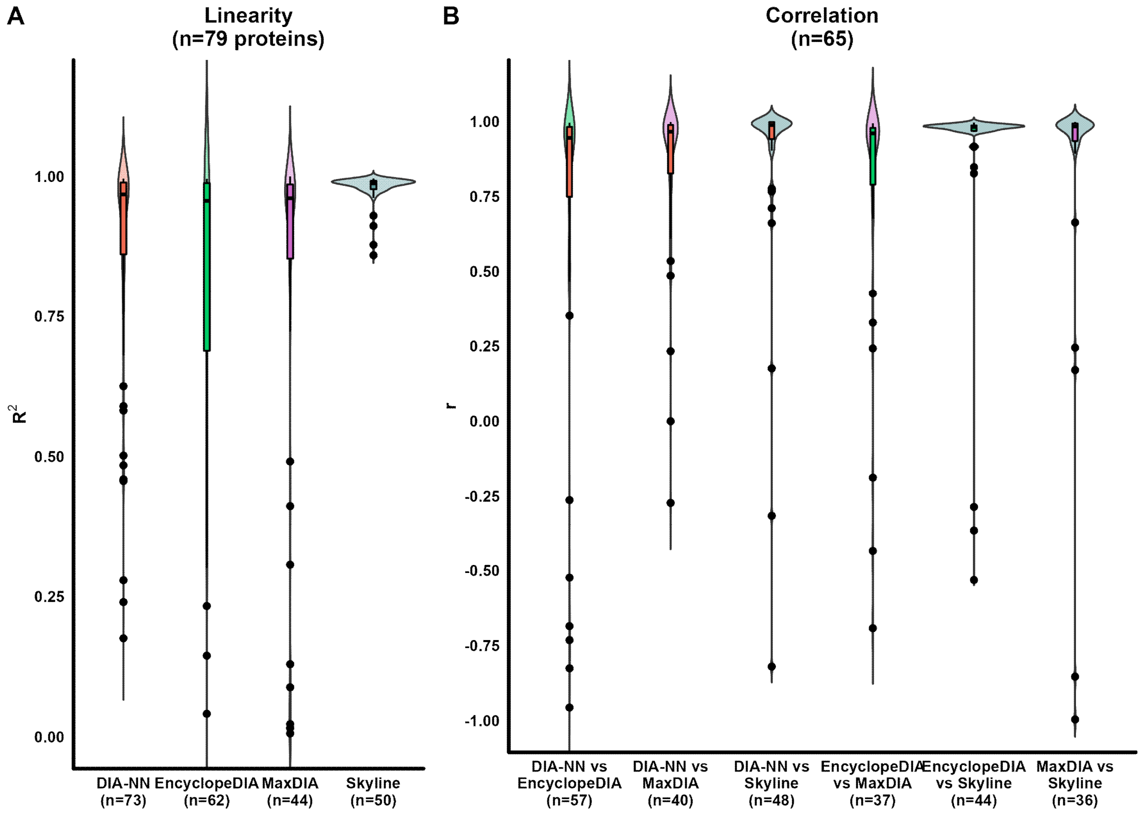


**Supplemental Figure S3. Assessment of linearity and correlation of HDL proteins diluted in *E. coli*.** Pooled HDL from six individuals was diluted in *E. coli* digest, ranging from 1 to 100% HDL in a total of 50 ng. The plots show 79 proteins quantified in two or more concentrations of HDL in at least one technical replicate across all four software. (A) Distribution of determination coefficients ($R^2$) of quadratic curves. (B) Distribution of Pearson correlation coefficients (r) for 65 proteins between all correlation pairs.

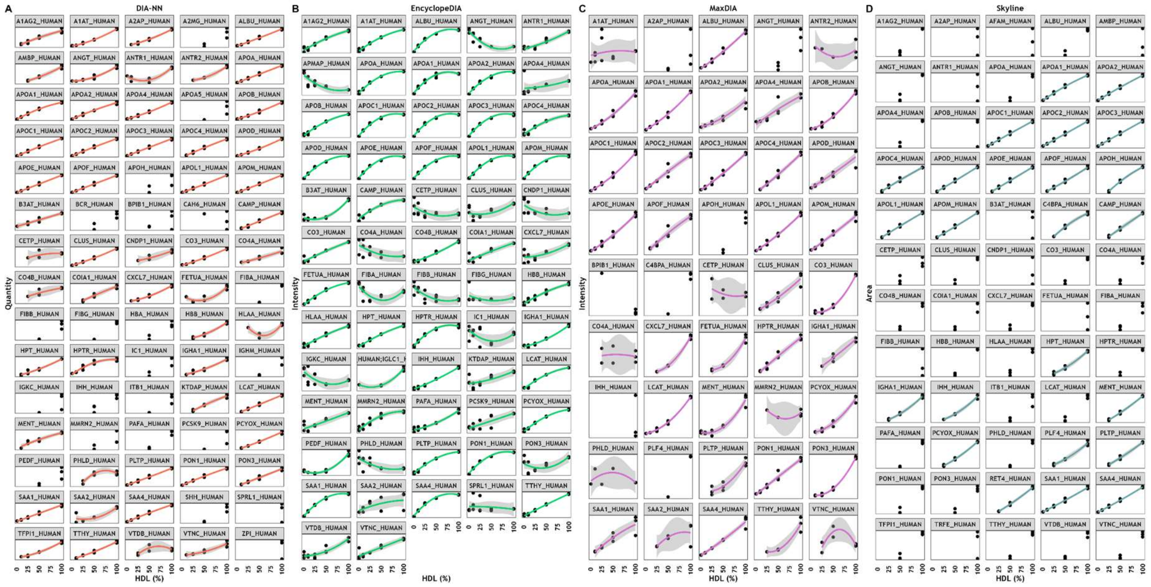


**Supplemental Figure S4. Individual assessment of linearity of HDL proteins diluted in *E. coli*.** Pooled HDL from six individuals was diluted in *E. coli* digest, ranging from 1 to 100% HDL in a total of 50 ng. The plots show all proteins quantified in two or more concentrations of HDL in at least one technical replicate across the software platforms: DIA-NN (A), EncyclopeDIA (B), MaxDIA (C) and Skyline (D). We evaluated linearity using a quadratic curve.

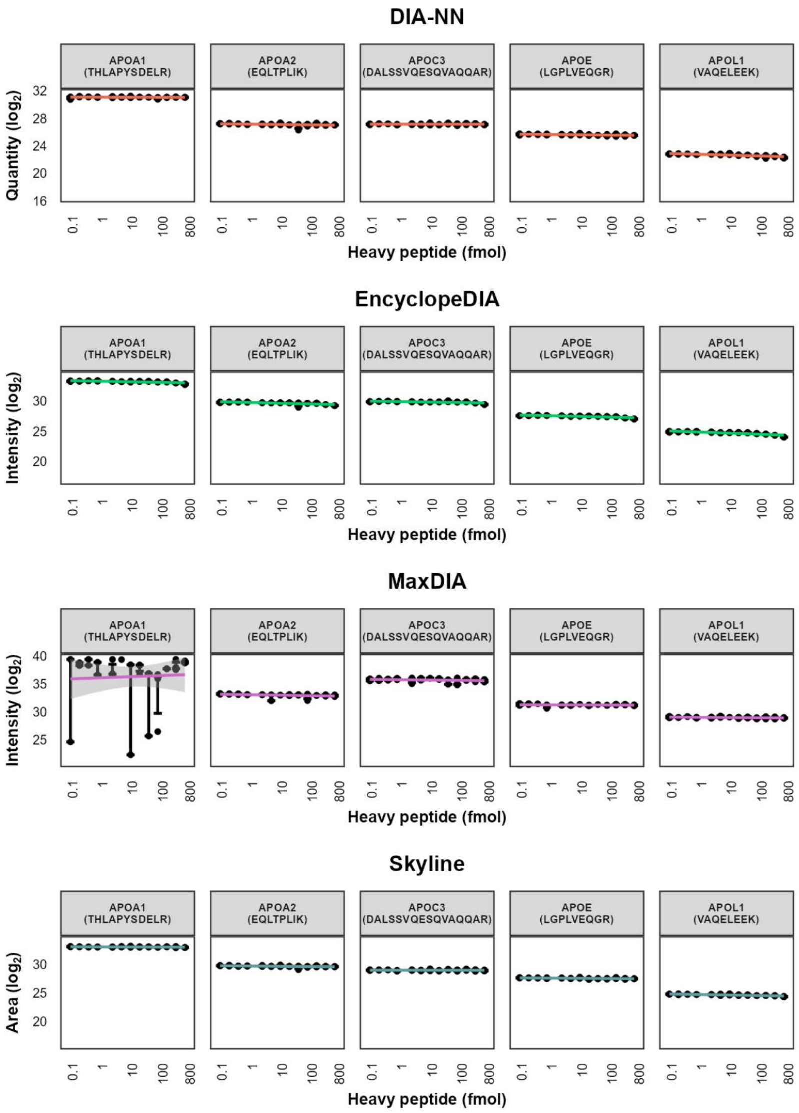


**Supplemental Figure S5. Representative plots of the technical variation obtained for the quantification of common HDL peptides.** Five HDL peptides belonging to APOA1, APOA2, APOC3, APOE and APOL1 were chosen for comparison with the variation in the area of standard curves obtained with labeled peptides in HDL (supplemental Figure S6 below). Intensity data were $\log_2$ normalized and peptides concentration was $\log_{10}$-scaled for visualization purposes.

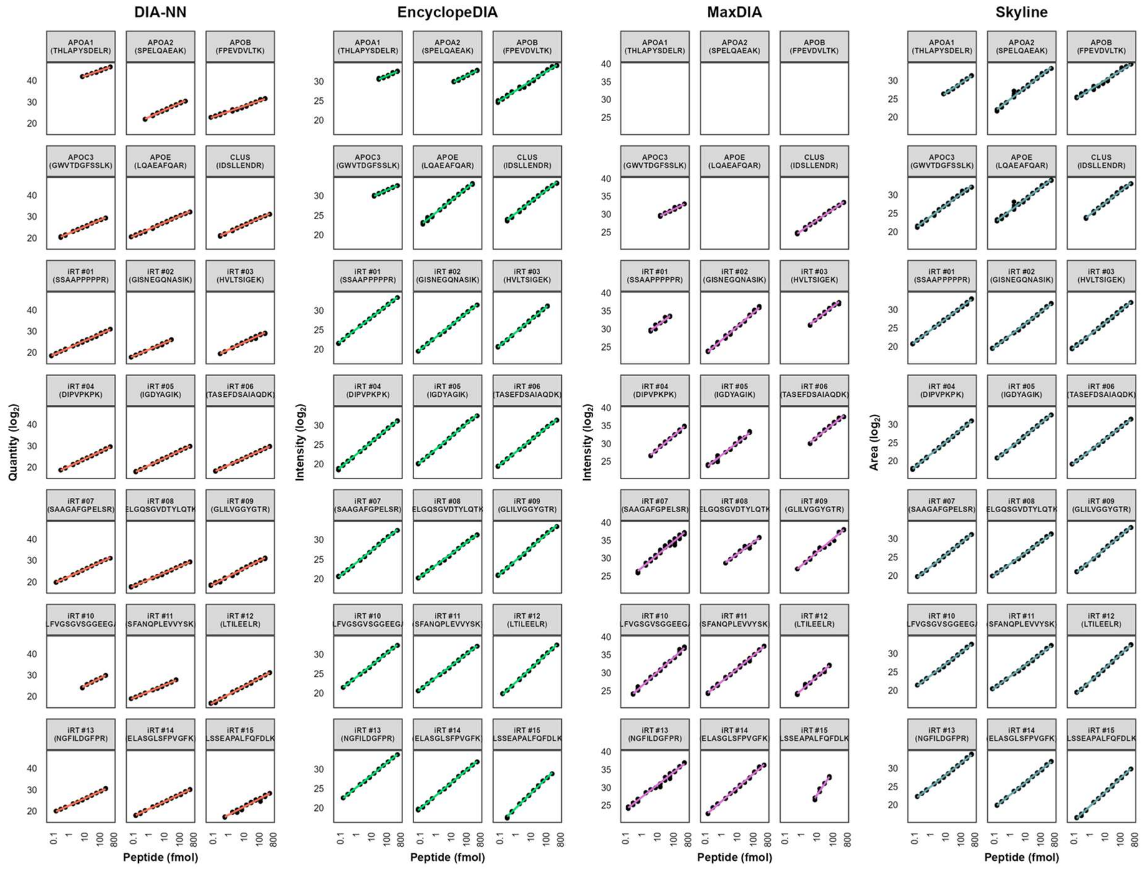


**Supplemental Figure S6. Individual plots of labeled peptides diluted in HDL**. Labeled peptides (from 0.125 to 800 fmol) were diluted in HDL (50 ng) and individual plots were constructed. Intensity data were $log_2$ normalized and peptides concentration was $log_{10}$-scaled for visualization purposes.

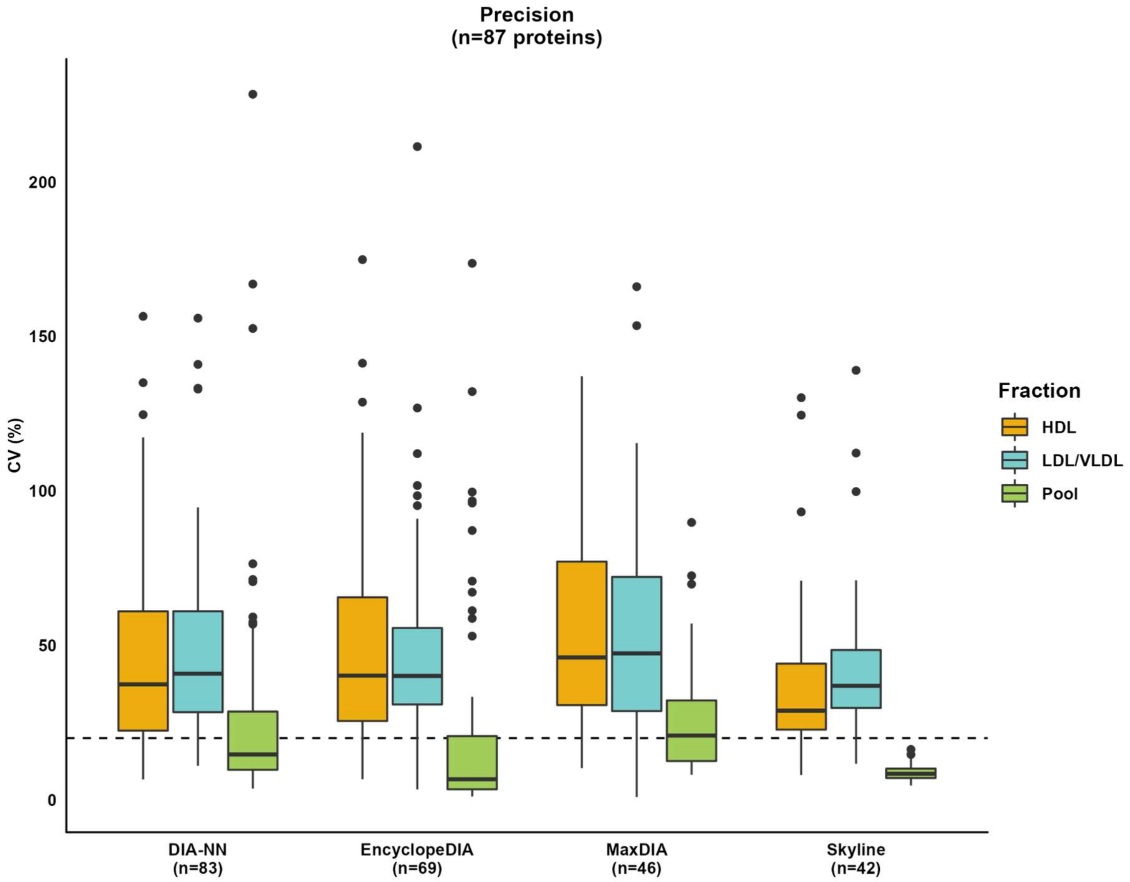


**Supplemental Figure S7. Comparison of technical and biological variability for all proteins quantified in HDL and LDL/VLDL.** HDL and LDL/VLDL were isolated from six apparently healthy individuals by two-step density ultracentrifugation. For comparison, a pooled sample was constructed from equal parts of each fraction from every individual, and injected 8 times. We used the four software to compare the precision of all proteins quantified by the software tools. A pooled HDL sample was used to assess technical variability.
